# Deep Learning/Artificial Intelligence and Blood-Based DNA Epigenomic Prediction of Cerebral Palsy

**DOI:** 10.3390/ijms20092075

**Published:** 2019-04-27

**Authors:** Ray O. Bahado-Singh, Sangeetha Vishweswaraiah, Buket Aydas, Nitish Kumar Mishra, Chittibabu Guda, Uppala Radhakrishna

**Affiliations:** 1 Department of Obstetrics and Gynecology, Oakland University William Beaumont School of Medicine, Royal Oak, MI 48073, USA; Ray.Bahado-Singh@beaumont.org (R.O.B.-S.); Sangeetha.Vishweswaraiah@beaumont.org (S.V.); 2 Department of Mathematics & Computer Science, Albion College, Albion, MI 49224, USA; baydas@albion.edu; 3 Dept. of Genetics, Cell Biology & Anatomy, College of Medicine, University of Nebraska Medical Center, Omaha, NE 68182, USA; nitish.mishra@unmc.edu (N.K.M.); babu.guda@unmc.edu (C.G.)

**Keywords:** DNA methylation, cerebral palsy, epigenetics, neurodegenerative disorders, newborns

## Abstract

The etiology of cerebral palsy (CP) is complex and remains inadequately understood. Early detection of CP is an important clinical objective as this improves long term outcomes. We performed genome-wide DNA methylation analysis to identify epigenomic predictors of CP in newborns and to investigate disease pathogenesis. Methylation analysis of newborn blood DNA using an Illumina HumanMethylation450K array was performed in 23 CP cases and 21 unaffected controls. There were 230 significantly differentially-methylated CpG loci in 258 genes. Each locus had at least 2.0-fold change in methylation in CP versus controls with a FDR *p*-value ≤ 0.05. Methylation level for each CpG locus had an area under the receiver operating curve (AUC) ≥ 0.75 for CP detection. Using Artificial Intelligence (AI) platforms/Machine Learning (ML) analysis, CpG methylation levels in a combination of 230 significantly differentially-methylated CpG loci in 258 genes had a 95% sensitivity and 94.4% specificity for newborn prediction of CP. Using pathway analysis, multiple canonical pathways plausibly linked to neuronal function were over-represented. Altered biological processes and functions included: neuromotor damage, malformation of major brain structures, brain growth, neuroprotection, neuronal development and de-differentiation, and cranial sensory neuron development. In conclusion, blood leucocyte epigenetic changes analyzed using AI/ML techniques appeared to accurately predict CP and provided plausible mechanistic information on CP pathogenesis.

## 1. Introduction

Cerebral palsy (CP) is a disorder of movement and posture that results from non-progressive injury to the developing brain [1,2]. The estimated prevalence of CP in the United States population is 3–4 cases per 1000 live births [3]. Cerebral white matter damage results in impaired motor development and control along with increased muscle tone and abnormal reflexes [4]. Associated co-morbidities in CP include attention deficit, disturbed perception and vision, epilepsy, intellectual function [5,6], and Autism Spectrum Disorders (ASD) [7]. Cerebral palsy is more frequently seen in males [8] and among black children compared to white children [9]. Most children diagnosed with CP have the spastic variety [10].

Cerebral palsy results from both genetic and environmental causes. Recognized etiological factors include viral and bacterial intrauterine infections, intrauterine growth restriction, antepartum hemorrhage, oxygen deprivation, placental complications, complicated and prenatal exposure to toxins among others [11]. This raises the possibility that CP could potentially be detected in the newborn period. The early diagnosis of CP remains a major clinical objective [12,13]. Early diagnosis permits intervention during critical periods of brain development and consequently can improve long term outcome. Currently, CP diagnosis is based on clinical history, physical exam, neuroimaging and genetic testing. The development of an effective laboratory tests could potentially represent an important advance in clinical care.

Epigenetic modification is now thought to be an important potential mechanism for prenatal brain injury leading to long-term motor, cognitive and behavioral dysfunction [14] and the potential benefits of epigenetic manipulation for CP therapy is now being recognized. The epigenetic basis of CP, however, remains to be more extensively investigated, however. Dysregulation of methylation capacity and folate single-carbon metabolism in children affected with severe CP [15] has been reported. Folate or single carbon metabolism provides the carbon substrate (methyl group) for DNA methylation, the most extensively studied epigenetic mechanism. DNA methylation is a well-recognized mechanism for control of gene transcription. A recent study of newborn blood spots found differential methylation of several CpG loci in monochorionic twins discordant for subsequent CP development [16].

In the current study, we performed global methylation profiling of CP cases and unaffected controls to identify significant methylation differences in CpG loci in leucocyte DNA. Differences in methylation levels at individual CpG loci between cases and controls were evaluated for the prediction of CP.

Artificial intelligence (AI) is a branch of computer sciences. Its objective is the development of machines whose cognitive functions related to problem solving exceed that of humans. Machine learning (ML) is a branch of AI [17] in which, using examples that are first provided, the computer develops its own logic for answering future questions. Given the large volume of data generated in epigenomic experiments, AI appears uniquely advantageous for analysis of OMIC studies. In omics including genomic studies AI appear to improve predictive model performance over alternative approaches [18]. Deep learning (DL) is the newest class of ML and has been found to be advantageous to other forms of ML [19,20]. DL employs multiple layers of neural networks, leading to expanded ‘neuronal’ complexity, to significantly enhance computational power. DL has been recently being applied to bioinformatics. We therefore evaluated DL and other forms of ML for the epigenomic prediction of CP. To our knowledge this has not been previously reported. Finally, we investigated the potential molecular pathogenesis of CP by focusing on the genes that were found to be epigenetically dysregulated.

## 2. Results

### 2.1. Identification of Differential Methylation between CP and Normal Controls

**Figure 1:**
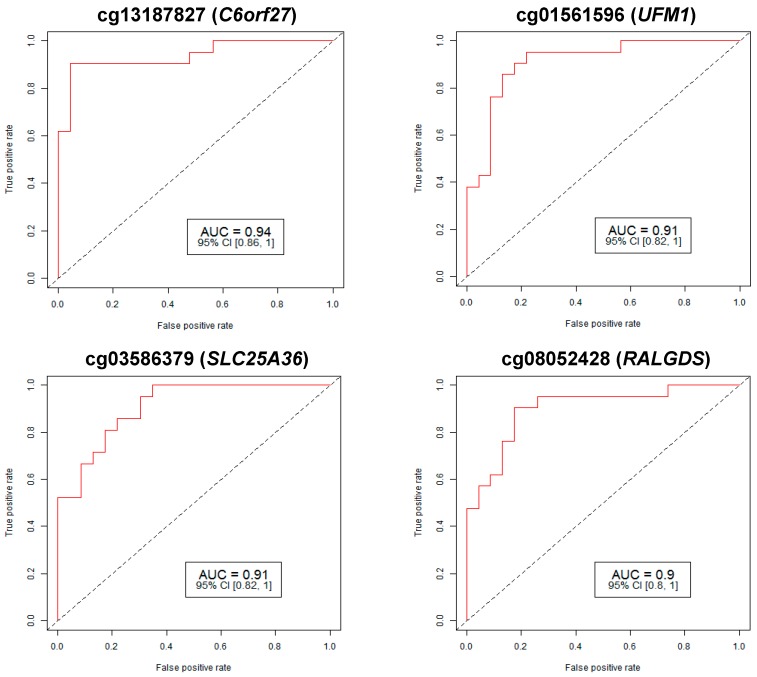
Receiver operating characteristic (ROC) curve analysis of methylation summaries for four specific markers linked with CP. The study identified 230 differentially-methylated CpG sites in 258 genes that have an area under the ROC curve ≥ 0.75 (*p*-value ≥ 0.05) for CP prediction. AUC: area under the receiver operating characteristics curve; 95% CI: 95% confidence interval. Lower and upper confidence intervals are given in parentheses.

We analyzed 23 leucocyte DNA samples from CP subjects and 21 from controls. Clinical comparisons between CP and controls are presented in Supplementary Table S1. There were no significant differences between the groups. A total of 15 CP cases had spastic CP (15/23= 65.2%). A total of 230 CpG loci from 258 genes that were found to be statistically significantly differentially-methylated in the CP false detection rate (FDR) *p*-value < 0.05 compared to controls (Supplementary Table S2). Apart from coding genes, we identified differentially-methylated CpGs in micro-RNA (miRNA), open reading frame genes (ORFs), non-coding RNA genes (NCRNAs), small nucleolar RNAs (SNOR), and LOC (unspecified) genes. Among them, the top 25 strongest individually predictive CpG loci are shown in Table 1. The area under the receiver operating curve (AUC-ROC) for four of the best performing individual CpG loci are shown in Figure 1. Overall, each individual locus had from moderate to high predictive accuracy for CP detection, AUC ≥ 0.75. A total of 128 CpG targets had AUC 0.80–0.89 (i.e., good predictor) and four targets had AUC ≥ 0.90 (i.e., excellent predictor).

**Figure 2:**
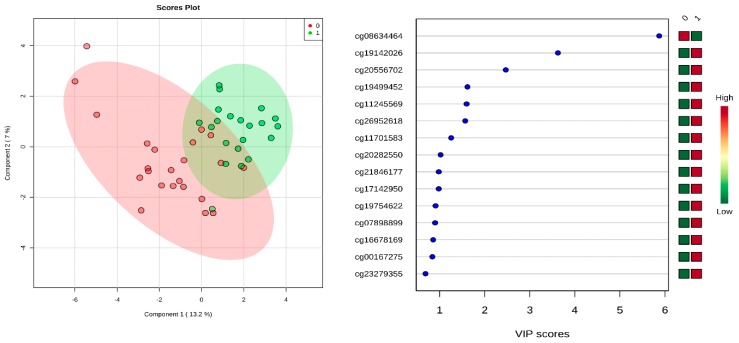
Two-dimensional partial least squares discriminant analysis (PLSDA-2D) of CP cases and control subjects. The red nodes (0) depict cases while the green nodes (1) represent controls.

Surprisingly, all 230 CpGs were hypomethylated in CP cases compared to controls at two-fold difference. CpGs were found to be distributed in gene body, 5′ UTR, 1st exon, transcription start site (TSS) 200, TSS1500 and 3’UTR. The PLS-DA plot shows separation of the two groups (Figure 2) using CpG loci having AUC (95% CI) of 0.97 (0.6–1) with a sensitivity of 0.90 and a specificity of 0.90. Permutation testing by 2000 cycles indicated that the separation was statistically significant (*p* = 0.012). The variable importance in projection (VIP) plot is also shown in Figure 2, it ranks predictive markers based on accuracy, with higher VIP score indicating greater predictive accuracy. We identified 12 DMRs with highly significant FDR *p*-value (Supplementary Table S3). Five of the DMRs were found to overlap with promoter regions. On conventional multivariate logistic regression, we obtained the model as follows: logit(P) = log(P/(1 − P)) = 0.153 + 3.713 cg01561596 − 1.897 cg12204727 + 0.148 cg17674287 + 0.798 cg20810398 + 1.904 cg16126458.

### 2.2. Newborn Prediction of Cerebral Palsy Using Deep Learning and Other ML Approaches

Using the top 230 most discriminating CpG loci (Table 1 and Supplementary Table S2) multiple ML techniques accurately predicted CP based on leucocyte methylation. DL was however a highly accurate predictor of CP with a sensitivity and specificity of 95% and 94.4%, respectively (Table 2) using a combination of CpG loci/genes. Finally, we identified a total of 42 genes (Fisher’s exact test *p*-value = 0.0001) in our dataset that contained CpG loci that were significantly differentially-methylated in genes that were previously reported to be differentially expressed in leucocytes of children with CP at 1.5 fold [21]. DL had a sensitivity and specificity of 96.4% and 90% respectively for CP detection (Supplementary Table S4) in this subgroup of genes. Overall, DL provided superior accuracy compared to other ML approaches.

### 2.3. Pathway and Network Analyses

**Figure 3:**
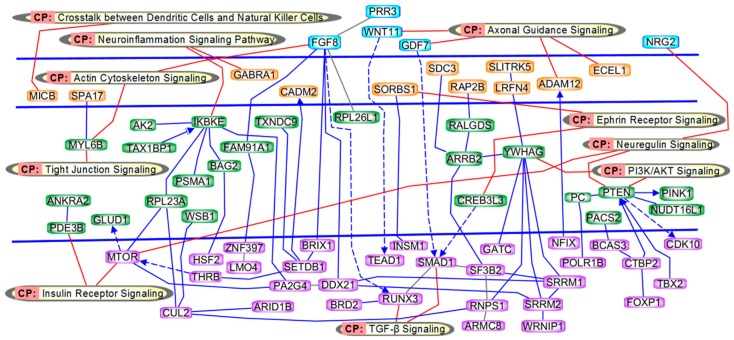
Ingenuity pathway analysis (IPA) results for 258 gene pathways included in the analysis. These genes were most highly differentially-methylated in association with CP. IPA results indicated the gene network are related to CP development, including: neuromotor damage, malformation of major brain structures, brain growth, neuroprotection, neuronal development and dedifferentiation, and cranial sensory neuron development.

Pathway and network analyses based on the differentially-methylated CpG loci and associated genes identified significant biological processes and functions related to the differentially-methylated genes (Supplementary Table S5 and Figure 3). Pathways included: Axonal guidance and actin cytoskeleton signaling, Wnt-signaling, insulin receptor and PI3K/AKT signaling, TGF-β signaling, crosstalk between dendritic cells and natural killer cells, neuroinflammation signaling pathway, ephrin receptor signaling, neuregulin signaling, and tight junction signaling. Genes previously known to be involved in brain function that were found to be differentially-methylated in our study included *ADAM12, FGF8, PTEN, PDE3B, SMAD1, RUNX3* as well as the gene for *miR*-1469.

On our methylation quantitative trait loci (mQTL) analysis, we observed one of the CpGs cg03586379 is a potential mQTL with a trans effect on the promoters at the time of birth. Other CpGs did not appear to be mQTLs at birth. We performed transcription factor binding site (TFBS) prediction for the top 4 predictors using ConTra v3 [22] and we determined that, only cg03586379 on the *SLC25A36* gene TSS200 has potential to be a transcription binding site. The other CpGs were not in currently identified TFBS or in the gene body region. Two transcripts of the *SLC25A36* gene, NM_001104647 and NM_018155 showed binding region for MA1047.1 (stringency: core = 0.95 and similarity matrix = 0.85) that has to be confirmed by further in vitro studies.

### 2.4. Validation by Pyrosequencing

The top 25 loci with the most significant changes were selected for independent validation by bisulfite pyrosequencing, based on their percentage differential methylation, AUC, fold change, and FDR p-values. These analyses revealed a high correlation between the results of the Illumina HumanMethylation450K BeadChip (San Diego, CA, USA) arrays data. We confirmed that the methylation status identified by the Illumina HumanMethylation450K arrays data was not biased but represented true changes.

## 3. Discussion

In this study, we identified significant epigenetic modification in leucocyte DNA of newborns who were subsequently diagnosed with CP. There were 230 significantly differentially-methylated CpG loci identified in CP compared to controls. These were associated with 258 genes. Early prediction of CP is crucial to improving long term outcomes and is the subject of much research efforts [23]. This was one important study objective. We therefore evaluated the potential utility of CpG methylation status for detection. Multiple individual loci with good to excellent predictive accuracy for CP detection were identified. Good predictive accuracy, defined as AUC ≥ 0.80–0.89 was found in 128 CpG loci while four CpG loci (genes), cg13187827 (*C6orf27*), cg01561596 (*UFM1*), cg03586379 (*SLC25A36*), and cg08052428 (*RALGDS*), had excellent predictive accuracy (AUC ≥ 0.90) for the detection of CP. Significant differences in cytosine methylation was observed not only in coding genes but also in miRNA genes, and genes for small nucleolar RNAs and other non-coding RNAs. 

Different AI-based machine learning (ML) techniques were evaluated for CP prediction based on CpG methylation status. Deep learning appeared consistently superior to the four other representative ML techniques used and achieved excellent predictive accuracy. For a specificity of 94.4%, sensitivities of 95% was achieved. The conventional multivariate logistic regression supports the ML prediction. The study of Mohandas et al. [16] found significant differential methylation in CpG loci of several genes in 15 monochorionic or ‘monozygotic’ twins, discordant for the later development of CP. This is consistent with our findings of significant epigenetic modifications found similar direction of methylation changes in few genes such as *PLOD2, C2orf47, AK2*, and *C2orf60.* The study by [16] did not, however, investigate whether epigenetic changes could function as screening tests for CP detection, an important objective of the current study. Our findings suggest the potential utility of epigenetic markers for newborn screening for CP.

A further objective of this study was to investigate the molecular pathogenesis of CP. Using the IPA analysis, a total of 69 genes were found to be involved in 10 canonical pathway mechanisms. The major canonical pathways with known significant relationship to brain function and a representative subgroup of important genes are discussed further.

### 3.1. Genes in Axonal Guidance and Actin Cytoskeleton Signaling

Axonal guidance is mainly mediated by Wnt proteins [24]. In cerebral cortex, the Wnt signaling regulates the migrating neurons [25]. Neuronal migration disruption occurs in several neurodevelopmental disorders including cerebral palsy [26]. Wnt proteins bind to the Frizzled transmembrane receptor to activate G proteins, which increase intracellular calcium levels, a cause of bone fragility. As a consequence, in children with cerebral palsy, disruption in bone homeostasis results in microdamage that, in turn, predisposes children to non-traumatic fractures [27]. Wnt proteins also play a major role in inducing Rho-dependent changes in the actin cytoskeleton [28]. Wingless-Type MMTV Integration Site Family, Member 11 (*WNT11)*, which belongs to the Wnt family of proteins, and *ADAM12* was found to be hypo-methylated in our study. *ADAM12* has a major role in reorganizing the actin cytoskeleton during early adipocyte differentiation [29]. Impairment of the actin cytoskeleton contributes to neuromotor damage, a pathogenic mechanism in cerebral palsy [30]. Fibroblast growth factor 8 (*FGF8*) was another hypo-methylated gene found in our study. The null mutation of this gene in mice confers lethality at an early embryonic stage and leads to malformation of major brain structures [31]. This indicates the importance of normal expression of these genes and suggests a potential pathogenic mechanism by which epigenetic disruption can lead to CP in our study population.

### 3.2. Genes in Insulin Receptor and PI3K/AKT Signaling

Impairment in serine/threonine phosphorylation of insulin receptor substrate proteins leads to insulin resistance, which could have pathophysiological implications in CP [32,33]. Phosphorylation impairment decreases binding of the downstream enzyme PI3K, altering the activation of Akt [33]. Akt is a kinase that inhibits apoptosis by phosphorylation of multiple apoptosis regulatory molecules and plays a crucial role in cell survival. Akt is upregulated in ischemia perfusion injuries of the brain and is the focus of significant clinical interest for the treatment of such injuries [34]. Ischemia is one of the major causes of brain injury associated with CP [35]. Interruptions in the interlinked insulin and PI3K/Akt signaling pathways may lead to significant brain effects in the case of CP. 

Phosphatase and tensin homolog *(PTEN)*, one of the differentially-methylated genes that we identified, is under PI3K/Akt influence and has been identified as an important molecule for promoting brain growth. *PTEN,* an epigenetically modified gene, plays a role in neuronal development and survival, synaptic plasticity and axonal regeneration and has been linked with neurodegenerative disorders [36,37]. *PDE3B* which is under the insulin receptor signaling and hypomethylated in our study, can combine with JAK2/PI3K pathways to play a neuroprotective role in the presence of G-CSF factor [38]. We also identified a hypomethylated pyruvate carboxylase gene (*PC*) in our study. PC is an active component of tricarboxylic acid (TCA) cycle that produces lactic acid [39]. Lactic acidosis has been linked to CP [40]. Epigenetic alteration of these complex interactions could plausibly play a role in CP pathogenesis.

### 3.3. Genes in TGF-β Signaling

TGF-β signaling plays a significant role in several neurodegenerative disorders. The pathway normally has neuroprotective properties including protection against excitotoxicity [41]. Neuronal TGF-β, is important for tissue regeneration, cell differentiation, and regulation of the immune system [42]. *SMAD1* has been implicated in neuronal development, differentiation and dedifferentiation [43]. SMAD proteins are intracellular signaling molecules that mediates the effect of TGFβ [44]. Runt-related transcription factor 3 (*RUNX3)*, regulates TGFβ signaling [45] and plays a crucial role in cranial sensory neuron development [46]. Both *SMAD1* and *RUNX3* were found to be hypo-methylated in the present study, and their involvement in anomalous neuronal development again makes a link between epigenetic dysregulation of critical neuronal genes and CP plausible. Of note, the study of Mohandas et al. (2018) on ‘monozygotic’ twins, discordant for the later development of CP, also found differential methylation of the leucocyte genes involved in TGF-β signaling, thus supporting the potential importance of epigenetic modification of TGF-β regulatory genes in CP.

### 3.4. miR-1469 in CP

MicroRNAs (miRNAs) are important in cell developmental processes like proliferation, differentiation, cell cycling and apoptosis. Along with these processes, miRNAs were also observed to play a role in neural cell patterning, establishment, plasticity, and neurogenesis [47,48]. We found the *miR-1469* gene to be significantly hypomethylated (FDR p-1.27 × 10^−8^) in CP. Differential expression of this gene has previously been observed in multiple neurological disorders [49,50,51,52,53] but to our knowledge had not been previously linked to CP.

### 3.5. Non-Coding RNAs and Small Nuclear RNAs

Non-coding RNAs (ncRNAs) do not code for proteins. The function of this group remains to be sufficiently elucidated, but they are thought to play a role in gene expression including epigenetic memory, transcription, translation, editing and RNA splicing [54,55]. Small nuclear RNAs (snRNA) is a member of the family of ncRNAs and is known to be involved in RNA biogenesis and stability, transcription, polyadenylation and eukaryotic gene expression [56]. We identified significant hypomethylation of a CpG locus in the TSS of *SNORD4A,* a snRNA, in the CP group versus controls. Two other ncRNAs *NCRNA00171* (gene body hypomethylation) and *NCRNA00028* (TSS hypomethylation) were also found to be significantly associated with CP in our study.

### 3.6. Limitations of the Study

While novel, our study does have limitations. A modest sample size was utilized. As this was exploratory using a modest sample size is practical. Despite the study size, we found highly significant methylation differences in a significant number of genes in CP cases. Although this was not our objective, another potential limitation of the study is that we were not able to do expression studies to see the correlation between the leucocyte gene methylation and changes in gene expression. Expression analysis was not feasible given that we utilized archived dried blood spots for this study. The expression profile is however an important issue. Thus, as an alternative approach, we searched leucocyte the expression database of van Eyk et al. [21]. Of the genes that they reported demonstrated differential expression in leucocytes in CP we identified 42 that in our study demonstrated statistically significant DNA methylation changes in newborns later diagnosed with CP compared to controls.

### 3.7. Conclusions

In conclusion, we identified significant epigenetic changes in multiple genes in leucocyte DNA of individuals diagnosed with CP. Early CP detection remains an important clinical objective. In the first approach of its kind we used AI techniques to accurately predict CP in newborns. We also identified molecular pathways which could mediate the development of CP, thus generating potentially important pathogenesis information. Larger validation studies would be an important next step.

## 4. Materials and Methods

Blood spots are routinely collected in Michigan for the newborn screening program for the detection of metabolic disorders. This program is run by the Michigan Department of Health and Human Services. After heel stick, blood spots were collected on filter paper between 24 and 79 hours after birth. Residual blood spots left over from clinically indicated screening are archived. Parents/legal guardians of the child provided informed written consent based on IRB approval from Wayne State University for medical chart review and to use residual blood spots for research purposes where available. The Michigan Department of Health and Human Services also provided IRB approval. The blood spot specimens were provided by the Michigan Department of Health and Human Services. Cases with suspected or known genetic syndromes or with congenital anomalies were excluded from this analysis.

### 4.1. Differential Methylation Assay

Leucocyte DNA was isolated from archived blood spots in 23 cases of CP and 21 controls using Puregene DNA Purification kits (Gentra systems^®^, Minneapolis, MN, USA) according to manufacturer’s protocols. The DNA samples were bisulfite converted using the EZ DNA Methylation-Direct Kit (Zymo Research, Orange, CA, USA) per the manufacturer’s protocol and processed according to Illumina protocols for HumanMethylation450K arrays.

### 4.2. Epigenome-Wide Methylation Scan Using Illumina Methylation Arrays

HumanMethylation450K arrays (San Diego, CA, USA). Genome wide methylation analysis was conducted on CP and control samples at 450,000 CpG loci. Cases and controls were performed in the same batch for analysis. Processing was done per manufacturer’s protocol [57]. Fluorescently-stained BeadChips were imaged by an Illumina iScan, following a series of stringent quality control and filtering criteria, as described previously [57].

### 4.3. Validation of Differential Methylation Analysis

We examined bisulfite-converted genomic DNA (EZ methylation kit by Zymo Research) by quantitative pyrosequencing analysis to confirm results from the Infinium Methylation arrays. Validation of methylation levels using pyrosequencing was performed on 20 cases and 15 controls. We performed pyrosequencing with appropriate oligos using the PyroMark Q24 System and advanced CpG Reagents (Qiagen^®^) as per the manufacturer’s instructions. We confirmed methylation difference of top 25 CpG targets, concluding the chip-based cytosine methylations are true changes [57,58]. A detailed methodology was published previously [57].

### 4.4. Statistical and Bioinformatic Analysis

The chi-square test of independence and equality of proportions for sample demographics were performed using SPSS tool. Bioinformatic and statistical analysis, data preprocessing and quality control were performed, including examination of the background signal intensity of both CP subjects and unaffected controls. DNA methylation was measured using the Genome Studio methylation analysis package (Illumina) including normalization. Subsequently, cytosine methylation levels or β-values were assigned to each CpG site. Potentially confounding factors such as probes associated with sex chromosomes and SNPs in the probe sequence (listing dbSNP entries within 10 bp of the CpG site) were removed for further analysis [59,60,61] as the nucleotide sequence may influence corresponding methylated probes [62]. Differential methylation was assessed by comparing the β-values per individual cytosine nucleotide at each measured CpG locus between cases and controls. We performed *t*-test (the difference between the mean of case and control) on individual CpG sites and calculated *p*-value and FDR *p*-value. Further, we used univariate logistic regression on individual CpG sites to calculate AUC (area under curve). Finally, we used FDR *p*-value ≤ 0.05 and AUC ≥ 0.75 to select differentially-methylated probes.

### 4.5. Partial Least Squares Discriminant Analysis (PLS-DA)

The PLS-DA distribution plot figure was performed by using MetaboAnalyst 4.0 [63] to determine whether CpG methylation could segregate CP group from controls. Data were subjected to sum normalization, log transformation and used multiple logistic regression statistics. Permutation testing was performed to confirm that any observed separation in the plot was statistically significant and not due to chance [63]. All CpG variables of CP cases and controls were computed together to detect variations between CP cases and controls. Variable Importance in Projection (VIP) scores were also used to rank predictors based on their contribution to discrimination of CP from normal controls. The higher the VIP score the better the predictor.

Pre-set criteria of ≥2.0-fold increase and/or ≥2.0-fold decrease and Benjamini-Hochberg False Discovery Rate (FDR) *p* < 0.05 for methylation difference were used to compare CP with controls. Individual CpG methylation level was used to calculate the area under the ROC curves (AUC) and 95% CI, sensitivity and specificity for AD detection. Area under the receiver operating characteristics curve (AUC) ≥ 0.75 for CP prediction were used to define significant methylation difference in CP compared to unaffected controls and this threshold suggests the potential for clinical utility as a predictor of CP.

In addition, we also used very stringent p-value thresholds (i.e., raw *p*-value < 5.0 × 10^−8^) to define significant methylation differences. This threshold is recommended for genome-wide analysis and is associated with reproducibility of the results [64]. We were unable to perform gene expression studies given the nature of the samples (dried blood spots). However, a prior study by van Eyk et al., [21] performed DNA expression analysis of leucocytes from children with CP compared to controls at 1.5 fold. We identified the genes that were differentially expressed in that study and cross matched these with genes (CpGs) that were also found to be significantly differentially-methylated in our study with the two-tailed Fisher’s exact test statistics.

### 4.6. Differentially-Methylated Regions (DMRs) Analysis

We have performed Differentially-methylated Region (DMR) analyses using Bioconductor tool *DMRcate* [65], this calculates differential methylation for individual CpG sites which is derivative of moderated *t*-statistic from *limma* [66] and subsequently FDR corrected significant dm-CpG regions were grouped where the distance between two consecutive probes is within 1 kb. Finally, we considered DMRs with minimum of two dm-CpGs that had an adjusted *p*-value < 0.01.

### 4.7. Logistic Regression with AUC (95% CI), Sensitivity and Specificity

A multiple logistic regression analysis was performed using stringent criteria (FDR *p* ≤0.001 and ≥2-fold change), to select an optimal combination of genes for CP prediction. We have used “GLM” package of “R” to perform logistic regression analysis.

### 4.8. Gene Ontology and Pathway Analysis

Significantly differentially-methylated CpG loci were utilized for further network and pathway analysis to help elucidate the pathogenesis of CP. Only genes for which Entrez identifiers were available were further analyzed. Gene ontology analysis and functional enrichment to identify dysregulated gene and gene-pathways in CP were performed, using QIAGEN’S Ingenuity Pathway Analysis Software to elucidate the mechanisms of isolated CP. Over-represented canonical pathways, biological processes and molecular processes were determined.

We also performed mQTL database search [67] to understand if any of our top 4 CpGs are strong mQTL and we performed Transcription factor binding site (TFBS) prediction for the top four predictors using ConTra v3 [22].

### 4.9. Artificial Intelligence (AI) Analysis Method Data Preprocessing

This approach is detailed in the Supplemental Methods section. Herein follows a summary of the analytic techniques. The descriptive methods on AI is provided as Supplementary Materials. In brief, each CpG β values were logged and auto scaled by its standard deviation. Quantile normalization was used to reduce sample-to-sample difference.

### 4.10. Deep Learning (DL)

To start, the first hidden layer (y) was activated by providing the sample input (x) to the first layer and deciding on the best parameters (W, b). Then, the second layer was predicted by utilizing the first hidden layer (y). The same process was repeated for all remaining layers-updating the weights and bias for each layer. Subsequently, we used back-propagation to regulate the parameters for all hidden layers. Finally, the Softmax classifier was used for the final hidden layer to assign new labels to the samples. We used the h2o R computer package to tune the parameters of the DL model [68,69].

### 4.11. Other Machine Learning Algorithms

In addition to DL we also evaluated a representative set of five machine learning (ML) algorithms which have been applied to data for classification and regression analyses [66]. The five models are, random forest (RF), support vector machine (SVM), linear discriminant analysis (LDA), prediction analysis for microarrays (PAM), and generalized linear model (GLM)—(logistic regression). To obtain the optimal predictive performance, we used the caret R computer package [70] to tune the parameters in the models.

### 4.12. Modeling and Evaluation

Outcomes prediction was based on methylation levels of CpG loci. Predictive accuracy was assessed based on area under the receiver-operating characteristic ROC curve (AUC 95% CI) along with sensitivity and specificity values. We randomly split the data into an 80% training set and the 20% as the test set. We performed 10-fold cross-validation (CV) on the 80% training data during the model construction process and tested the model on the hold out 20% of data. We used the R package, pROC, to compute AUC of ROC to assess the overall performance of the models.

### 4.13. CP Prediction Based on AI Analysis

The AUC (95% CI), sensitivity and specificity were calculated based on top 240 best performing individual CpG loci (based on individual AUC, fold-change in methylation and absolute percentage methylation difference and FDR *p*-value for CP versus controls). This was repeated using only the 76 individual loci that exceeded the high stringency threshold, i.e., *p*-value < 5 × 10^−8^. 

Finally, as noted previously a prior publication identified leucocyte genes that are differentially expressed in CP cases [21]. We identified those published differentially expressed genes that also had significantly differentially-methylated CpG loci in our study. Using the single best individual performing loci (i.e., for distinguishing CP case from controls) per gene, we employed AI techniques to determine the optimal combination of CpG loci (from multiple genes) for CP detection.

The following parameters were used to tune the DL model:

- Epochs (number of passes of the full training set),
- l1 (penalty to converge the weights of the model to 0),
- l2 (penalty to prevent the enlargement of the weights),
- Input dropout ratio (ratio of ignored neurons in the input layer during training),
- Number of hidden layers;

The parameters that were used to tune the SVM model was the cost of classification; to tune the RF model was the number of trees to fit, and to tune the PAM model was the threshold amount for shrinking toward the centroid.

### 4.14. Overfitting and Computation Time

To avoid overfitting in the DL model, we used three regularization parameters: L1, which increases model stability and causes many weights to become 0 and L2, which prevents weight enlargement, while L2 prevents any single weight from getting very large values. The third parameter that we used for avoiding overfitting in DL model was the input dropout ratio which controls the amount of input layer neurons that are randomly dropped (set to zero) and controls overfitting with respect to the input data. This is particularly useful for high-dimensional noisy data [71]. 

### 4.15. Feature Importance

Feature (predictor) importance was estimated using a model-based approach. We used the variable importance functions in h2o (varimp) and in caret R packages (varimp) to rank the models features in each of the predictive algorithms [69].

## Figures and Tables

**Table 1:** Details of top 25 CpG targets significantly differentially-methylated in CP based on AUC. Target ID, Gene ID, chromosome location,% methylation change, and FDR *p*-value are provided.

Target ID	Chr	Gene	FDR *p*-Val	Fold Change	% Methylation		AUC	CI	
					Cases	Control		Lower	Upper
cg13187827	6	C6orf27	4.56 × 10^-28^	0.47	12.88	27.47	0.94	0.86	1
cg01561596	13	UFM1	0.00296	0.43	1.57	3.67	0.91	0.82	1
cg03586379	3	SLC25A36	1.02 × 10^−5^	0.41	2.33	5.64	0.91	0.82	1
cg08052428	9	RALGDS	1.53 × 10^−8^	0.48	4.66	9.63	0.90	0.8	1
cg07898899	1	S100A13	3.72 × 10^−20^	0.42	7.11	16.87	0.89	0.79	0.99
cg17142950	1	SAMD13	1.33 × 10^−30^	0.44	12.21	27.61	0.88	0.77	0.98
cg20376421	12	MYL6B	4.40 × 10^−7^	0.49	4.14	8.41	0.88	0.78	0.99
cg10230427	6	BAG2	6.70 × 10^−12^	0.41	4.22	10.24	0.87	0.76	0.98
cg14347670	6	CCND3	5.68 × 10^−8^	0.4	2.81	7.07	0.87	0.75	0.98
cg20640432	19	CREB3L3	0.00015	0.5	2.91	5.86	0.87	0.75	0.98
cg00472801	6	KHDRBS2	8.40 × 10^−7^	0.5	4.08	8.23	0.86	0.74	0.97
cg03307401	19	KLK13	0.00017	0.36	1.45	4.09	0.86	0.74	0.97
cg11961138	17	IGFBP4	2.48 × 10^−21^	0.39	6.14	15.87	0.86	0.74	0.97
cg12204727	15	COMMD4	0.02176	0.5	1.63	3.27	0.86	0.75	0.97
cg12206423	13	SLITRK5	0.00012	0.49	2.91	5.9	0.86	0.74	0.97
cg17852224	22	MAPK8IP2	1.45 × 10^−11^	0.47	5.51	11.83	0.86	0.74	0.97
cg20871904	4	YTHDC1	3.95 × 10^−5^	0.47	2.75	5.92	0.86	0.74	0.97
cg26707202	4	SMAD1	1.68 × 10^−6^	0.42	2.66	6.35	0.86	0.74	0.97
cg01067849	6	WRNIP1	0.00058	0.42	1.76	4.23	0.85	0.73	0.97
cg02782426	3	ENTPD3	1.94 × 10^−7^	0.47	3.9	8.26	0.85	0.74	0.97
cg03433549	12	PA2G4	0.00456	0.47	1.86	3.91	0.85	0.73	0.97
cg08931196	11	RNF26	0.03450	0.47	1.33	2.81	0.85	0.73	0.97
cg15277906	8	GDF6	0.00073	0.5	2.5	5.05	0.85	0.73	0.97
cg20810398	1	EXOSC10	0.04950	0.48	1.27	2.64	0.85	0.73	0.97
cg22624212	21	WDR4	0.00137	0.43	1.75	4.04	0.85	0.73	0.97

**Table 2:** Results of CP AI/DL predictions based on the top 230 individual CpG loci.

	SVM	GLM	PAM	RF	LDA	DL
**AUC 95% CI**	0.9875 (0.6875–1)	0.9765 (0.6765–1)	0.8468 (0.6468–1)	0.9087 (0.6087–1)	0.9675 (0.6675–1)	0.9760 (0.6760–1)
**Sensitivity**	0.9200	0.8500	0.7500	0.7500	0.8000	0.9500
**Specificity**	0.9200	0.8500	0.9000	0.9000	0.9000	0.9440

Important predictors in descending order: SVM: cg13187827, cg01561596, cg07898899, cg12204727, cg03586379; GLM: cg01561596, cg12204727, cg17674287, cg20810398, cg16126458; PAM: cg13187827, cg08052428, cg01561596, cg03586379, cg18516195; RF: cg13187827, cg01561596, cg20640432, cg14347670, cg07898899; LDA: cg13187827, cg01561596, cg20640432, cg07898899, cg03586379; DL: cg01561596, cg12425861, cg13187827, cg12204727, cg03586379.

## Supplementary Materials

Supplementary Materials can be found at https://www.mdpi.com/1422-0067/20/9/2075/s1.
