# Supplementary material for: Deep Learning/Artificial Intelligence and Blood-Based DNA Epigenomic Prediction of Cerebral Palsy
Source: Int J Mol Sci. 2019 Apr 27;20(9):2075. doi: 10.3390/ijms20092075 (PMC6539236; doi:10.3390/ijms20092075)
Supplement: Supplementary file 1 [file ijms-20-02075-s001.zip › ijms-437963-supplementary/4-CP-Supplementary Table S1.docx]

**Supplementary Table S1.** Comparison of demographics and clinical characteristics: cerebral Palsy cases vs. controls.

| **Parameter** | **CP Cases** | **Controls** | ***p*-value** |
| --- | --- | --- | --- |
| Number of patients | 23 | 21 |  |
| Age in weeks -  Mean (Standard deviation) | 37.740 (2.61) | 37.590 (3.11) | 0.86 |
| Gender | 0.65 (0.49) | 0.57 (0.51) | 0.75 |
| Race |  |  | 0.67* |
| - White—N (%) | 12 (52.17) | 11 (52.38) |  |
| - African American—N (%) | 7 (30.40) | 7 (33.33) |  |
| - Hispanic—N (%) | 4 (17.43) | 3 (14.29) |  |

*Chi square
