# Supplementary material for: Deep Learning/Artificial Intelligence and Blood-Based DNA Epigenomic Prediction of Cerebral Palsy
Source: Int J Mol Sci. 2019 Apr 27;20(9):2075. doi: 10.3390/ijms20092075 (PMC6539236; doi:10.3390/ijms20092075)
Supplement: Supplementary file 1 [file ijms-20-02075-s001.zip › ijms-437963-supplementary/5-CP-Supplementary Table S2.docx]

**Supplementary Table S2.** CpG loci that were differentially methylated in cerebral palsy arranged based on AUC. Target ID, Gene ID, chromosome location, % methylation levels in CP and controls, AUC (95% CI), and FDR p-value for CP prediction.

| **Target ID** | **Chr** | **Gene** | **FDR p-Val** | **Fold change** | **% Methylation** | | **AUC** | **CI** | |
| --- | --- | --- | --- | --- | --- | --- | --- | --- | --- |
|  |  |  |  |  | **Cases** | **Control** |  | **Lower** | **Upper** |
| cg24069733 | 20 | DBNDD2; SYS1 | 0.00107 | 0.43 | 1.75 | 4.09 | 0.85 | 0.73 | 0.97 |
| cg26952618 | 16 | FAM18A | 1.97E-21 | 0.32 | 4.05 | 12.87 | 0.85 | 0.73 | 0.97 |
| cg01712673 | 17 | WBP2 | 0.00635 | 0.49 | 1.93 | 3.91 | 0.84 | 0.72 | 0.96 |
| cg03309770 | 16 | FAM18A | 1.80E-10 | 0.48 | 5.6 | 11.55 | 0.84 | 0.72 | 0.96 |
| cg05332869 | 20 | TOP1 | 0.00016 | 0.49 | 2.78 | 5.69 | 0.84 | 0.72 | 0.96 |
| cg08171351 | 22 | CECR6 | 2.99E-08 | 0.49 | 4.57 | 9.41 | 0.84 | 0.72 | 0.96 |
| cg10333402 | 7 | MOGAT3 | 5.14E-09 | 0.49 | 5.08 | 10.35 | 0.84 | 0.72 | 0.96 |
| cg16126458 | 1 | AKR7A3 | 2.06E-05 | 0.45 | 2.66 | 5.92 | 0.84 | 0.71 | 0.96 |
| cg17486946 | 10 | FGF8 | 7.20E-07 | 0.45 | 3.33 | 7.32 | 0.84 | 0.72 | 0.96 |
| cg17674287 | 6 | BRD2 | 0.03636 | 0.47 | 1.28 | 2.74 | 0.84 | 0.72 | 0.96 |
| cg18132212 | 4 | NSUN7 | 0.01680 | 0.43 | 1.26 | 2.92 | 0.84 | 0.72 | 0.96 |
| cg18384060 | 10 | PTEN; KILLIN | 0.01669 | 0.46 | 1.46 | 3.15 | 0.84 | 0.72 | 0.96 |
| cg18516195 | 14 | BEGAIN | 8.54E-28 | 0.45 | 11.68 | 25.73 | 0.84 | 0.72 | 0.96 |
| cg19917744 | 2 | PLEKHM3 | 8.95E-07 | 0.39 | 2.32 | 6.02 | 0.84 | 0.72 | 0.96 |
| cg23000734 | 10 | CTBP2 | 1.40E-18 | 0.46 | 8.08 | 17.71 | 0.84 | 0.72 | 0.96 |
| cg00114084 | 1 | AK2 | 0.01595 | 0.41 | 1.16 | 2.83 | 0.83 | 0.7 | 0.95 |
| cg00167275 | 10 | FAM35A; GLUD1 | 1.73E-18 | 0.46 | 8 | 17.57 | 0.83 | 0.7 | 0.95 |
| cg01158970 | 5 | UTP15; ANKRA2 | 0.00343 | 0.46 | 1.82 | 3.93 | 0.83 | 0.7 | 0.95 |
| cg02553987 | 17 | BCAS3 | 0.02526 | 0.46 | 1.32 | 2.88 | 0.83 | 0.7 | 0.95 |
| cg02743650 | 11 | IGSF22 | 3.97E-06 | 0.5 | 3.8 | 7.61 | 0.83 | 0.7 | 0.95 |
| cg04527840 | 4 | GAR1 | 0.01419 | 0.42 | 1.22 | 2.92 | 0.83 | 0.7 | 0.95 |
| cg05359249 | 2 | CHPF | 0.01615 | 0.39 | 1.05 | 2.69 | 0.83 | 0.71 | 0.96 |
| cg06106763 | 21 | OLIG1 | 0.00030 | 0.3 | 1.06 | 3.53 | 0.83 | 0.7 | 0.95 |
| cg07162198 | 20 | SLC2A10 | 0.00719 | 0.49 | 1.88 | 3.83 | 0.83 | 0.7 | 0.95 |
| cg08301299 | 16 | RNPS1 | 3.08E-09 | 0.41 | 3.29 | 8.13 | 0.83 | 0.71 | 0.96 |
| cg08979136 | 5 | TRIM36 | 0.03987 | 0.45 | 1.14 | 2.57 | 0.83 | 0.7 | 0.95 |
| cg09883524 | 16 | MC1R | 0.01450 | 0.47 | 1.53 | 3.27 | 0.83 | 0.71 | 0.96 |
| cg10384919 | 22 | MEI1 | 1.06E-08 | 0.47 | 4.5 | 9.49 | 0.83 | 0.7 | 0.95 |
| cg10760299 | 15 | GATM | 6.44E-15 | 0.5 | 8.32 | 16.75 | 0.83 | 0.7 | 0.95 |
| cg11722376 | 2 | LOC389033 | 1.89E-16 | 0.47 | 7.81 | 16.61 | 0.83 | 0.71 | 0.95 |
| cg13331200 | 3 | CADM2 | 4.96E-07 | 0.41 | 2.74 | 6.65 | 0.83 | 0.7 | 0.95 |
| cg13404674 | 12 | IQSEC3 | 2.49E-28 | 0.5 | 24.55 | 49.45 | 0.83 | 0.7 | 0.95 |
| cg15953602 | 8 | CRISPLD1 | 0.00317 | 0.49 | 2.07 | 4.24 | 0.83 | 0.7 | 0.95 |
| cg17009717 | 2 | POLR1B | 0.01885 | 0.49 | 1.64 | 3.32 | 0.83 | 0.71 | 0.95 |
| cg18426487 | 10 | CUL2 | 0.00490 | 0.45 | 1.65 | 3.66 | 0.83 | 0.71 | 0.96 |
| cg19243130 | 11 | SIAE; SPA17 | 0.00074 | 0.46 | 2.08 | 4.56 | 0.83 | 0.71 | 0.96 |
| cg20677058 | 1 | AKR7L | 2.38E-11 | 0.42 | 4.15 | 9.97 | 0.83 | 0.71 | 0.96 |
| cg00003287 | 1 | TNNT2 | 3.31E-05 | 0.46 | 2.72 | 5.9 | 0.82 | 0.7 | 0.95 |
| cg01572696 | 4 | IDUA | 1.21E-11 | 0.49 | 6.44 | 13.08 | 0.82 | 0.7 | 0.95 |
| cg02678768 | 17 | EVPL | 6.64E-29 | 0.47 | 19.75 | 42.11 | 0.82 | 0.69 | 0.95 |
| cg05389183 | 5 | PPIC | 8.68E-09 | 0.48 | 4.62 | 9.67 | 0.82 | 0.7 | 0.95 |
| cg06463589 | 16 | MT1E | 0.01569 | 0.48 | 1.61 | 3.34 | 0.82 | 0.69 | 0.95 |
| cg06951245 | 2 | PTH2R | 1.02E-05 | 0.45 | 2.78 | 6.19 | 0.82 | 0.69 | 0.95 |
| cg11156873 | 5 | LPCAT1 | 1.88E-30 | 0.45 | 13.17 | 29.16 | 0.82 | 0.7 | 0.95 |
| cg12721730 | 13 | PCDH20 | 2.80E-07 | 0.46 | 3.59 | 7.8 | 0.82 | 0.69 | 0.95 |
| cg13206850 | 7 | ATXN7L1 | 2.68E-29 | 0.5 | 20.64 | 41.31 | 0.82 | 0.69 | 0.95 |
| cg13717541 | 14 | CLMN | 5.38E-28 | 0.47 | 23.05 | 49.48 | 0.82 | 0.69 | 0.95 |
| cg13931999 | 9 | HINT2 | 0.00368 | 0.45 | 1.66 | 3.73 | 0.82 | 0.69 | 0.95 |
| cg14621053 | 10 | ADAM12 | 0.02035 | 0.48 | 1.51 | 3.16 | 0.82 | 0.69 | 0.95 |
| cg22476848 | 22 | MEI1 | 1.03E-06 | 0.43 | 2.82 | 6.63 | 0.82 | 0.69 | 0.95 |
| cg25302370 | 6 | C6orf165 | 0.02907 | 0.5 | 1.55 | 3.11 | 0.82 | 0.69 | 0.95 |
| cg01534217 | 3 | FOXP1 | 0.00020 | 0.39 | 1.7 | 4.36 | 0.81 | 0.67 | 0.94 |
| cg01802975 | 1 | SLC35D1 | 0.00016 | 0.5 | 2.86 | 5.78 | 0.81 | 0.68 | 0.94 |
| cg02970551 | 1 | RUNX3 | 7.60E-08 | 0.44 | 3.41 | 7.78 | 0.81 | 0.68 | 0.94 |
| cg09605254 | 8 | FAM91A1 | 2.59E-08 | 0.43 | 3.37 | 7.9 | 0.81 | 0.68 | 0.94 |
| cg10241347 | 10 | FAM24B; LOC399815 | 1.07E-15 | 0.43 | 5.78 | 13.6 | 0.81 | 0.68 | 0.94 |
| cg10961700 | 1 | SETDB1 | 0.00191 | 0.5 | 2.27 | 4.57 | 0.81 | 0.68 | 0.94 |
| cg11701583 | 12 | NDUFA4L2 | 7.02E-29 | 0.42 | 9.75 | 23.37 | 0.81 | 0.68 | 0.94 |
| cg14234406 | 8 | PLEC1 | 4.27E-17 | 0.44 | 6.61 | 15.19 | 0.81 | 0.68 | 0.94 |
| cg16565409 | 17 | RPL23A; SNORD4A | 2.48E-29 | 0.43 | 15.66 | 36.19 | 0.81 | 0.67 | 0.94 |
| cg16579438 | 3 | THRB | 1.54E-07 | 0.43 | 3.13 | 7.31 | 0.81 | 0.68 | 0.94 |
| cg16741308 | 22 | PARVB | 0.03333 | 0.46 | 1.26 | 2.75 | 0.81 | 0.68 | 0.94 |
| cg16783819 | 6 | HSF2 | 0.00123 | 0.47 | 2.13 | 4.51 | 0.81 | 0.68 | 0.94 |
| cg16857181 | 7 | KBTBD2 | 0.00042 | 0.48 | 2.46 | 5.12 | 0.81 | 0.68 | 0.94 |
| cg17012160 | 1 | FMN2 | 2.43E-06 | 0.46 | 3.19 | 6.94 | 0.81 | 0.68 | 0.94 |
| cg19226007 | 17 | C1QL1 | 0.00233 | 0.44 | 1.73 | 3.91 | 0.81 | 0.68 | 0.94 |
| cg20282550 | 10 | AKR1E2 | 9.30E-13 | 0.36 | 3.41 | 9.42 | 0.81 | 0.68 | 0.94 |
| cg21144587 | 2 | GPN1; CCDC121 | 1.86E-11 | 0.49 | 6.36 | 12.9 | 0.81 | 0.68 | 0.94 |
| cg21558545 | 12 | LGR5 | 0.00022 | 0.46 | 2.31 | 5.07 | 0.81 | 0.68 | 0.94 |
| cg21846177 | 20 | NCRNA00028 | 1.83E-16 | 0.35 | 4.02 | 11.38 | 0.81 | 0.67 | 0.94 |
| cg22130262 | 8 | MOS | 0.00018 | 0.41 | 1.87 | 4.58 | 0.81 | 0.68 | 0.94 |
| cg22284043 | 13 | GPC5 | 0.00067 | 0.5 | 2.58 | 5.16 | 0.81 | 0.67 | 0.94 |
| cg22704520 | 2 | C2orf47; C2orf60 | 9.52E-09 | 0.49 | 5.02 | 10.16 | 0.81 | 0.68 | 0.94 |
| cg22803211 | 4 | OCIAD1 | 0.02382 | 0.48 | 1.47 | 3.07 | 0.81 | 0.67 | 0.94 |
| cg23279355 | 5 | CMYA5 | 2.94E-25 | 0.45 | 10.71 | 23.56 | 0.81 | 0.68 | 0.94 |
| cg23731836 | 8 | KIF13B | 0.00447 | 0.47 | 1.81 | 3.86 | 0.81 | 0.68 | 0.94 |
| cg26273962 | 10 | SORBS1 | 0.04706 | 0.36 | 0.75 | 2.08 | 0.81 | 0.68 | 0.94 |
| cg00788028 | 2 | LOC440839 | 1.09E-12 | 0.47 | 6.23 | 13.18 | 0.8 | 0.66 | 0.93 |
| cg03380643 | 20 | INSM1 | 0.02585 | 0.49 | 1.52 | 3.1 | 0.8 | 0.66 | 0.93 |
| cg04582164 | 3 | RAP2B | 0.00474 | 0.5 | 2.07 | 4.15 | 0.8 | 0.67 | 0.94 |
| cg05044431 | 5 | GABRA1 | 0.01286 | 0.46 | 1.5 | 3.26 | 0.8 | 0.67 | 0.93 |
| cg06508976 | 9 | IER5L | 0.00043 | 0.43 | 1.91 | 4.46 | 0.8 | 0.67 | 0.94 |
| cg06604058 | 11 | RTN3 | 1.52E-09 | 0.46 | 4.5 | 9.8 | 0.8 | 0.66 | 0.93 |
| cg07755735 | 2 | GDF7 | 4.65E-13 | 0.48 | 6.81 | 14.08 | 0.8 | 0.67 | 0.93 |
| cg08019195 | 11 | SCN4B | 0.01818 | 0.46 | 1.44 | 3.11 | 0.8 | 0.67 | 0.93 |
| cg08326511 | 2 | DBI; C2orf76 | 0.03057 | 0.48 | 1.4 | 2.92 | 0.8 | 0.67 | 0.93 |
| cg08928494 | 16 | CA5A | 7.04E-29 | 0.46 | 18.86 | 41.33 | 0.8 | 0.67 | 0.93 |
| cg10301338 | 18 | KCTD1 | 0.00854 | 0.46 | 1.61 | 3.49 | 0.8 | 0.67 | 0.94 |
| cg11688874 | 10 | WAC | 9.11E-07 | 0.43 | 2.92 | 6.77 | 0.8 | 0.66 | 0.93 |
| cg11796565 | 19 | NFIX | 8.83E-06 | 0.47 | 3.04 | 6.53 | 0.8 | 0.67 | 0.94 |
| cg11963883 | 10 | DDX21 | 0.01154 | 0.33 | 0.83 | 2.52 | 0.8 | 0.67 | 0.93 |
| cg18382422 | 10 | TSPAN15 | 0.00358 | 0.47 | 1.86 | 3.97 | 0.8 | 0.67 | 0.94 |
| cg19142026 | 7 | HOXA4 | 3.49E-25 | 0.3 | 4.16 | 14.06 | 0.8 | 0.66 | 0.93 |
| cg19580633 | 5 | RPL26L1; LOC100268168 | 0.00306 | 0.42 | 1.48 | 3.56 | 0.8 | 0.67 | 0.93 |
| cg19737664 | 11 | LRRC56 | 4.22E-06 | 0.46 | 3.14 | 6.79 | 0.8 | 0.66 | 0.93 |
| cg20914370 | 7 | TAX1BP1 | 0.01277 | 0.34 | 0.87 | 2.55 | 0.8 | 0.67 | 0.93 |
| cg21914984 | 2 | CDC42EP3 | 0.00112 | 0.45 | 1.93 | 4.3 | 0.8 | 0.66 | 0.93 |
| cg22028544 | 8 | C8orf59 | 0.03358 | 0.37 | 0.84 | 2.28 | 0.8 | 0.66 | 0.93 |
| cg22363327 | 6 | SFRS13B | 2.58E-09 | 0.49 | 5.3 | 10.74 | 0.8 | 0.66 | 0.93 |
| cg24441627 | 12 | BRI3BP | 0.01067 | 0.43 | 1.36 | 3.15 | 0.8 | 0.66 | 0.93 |
| cg24455365 | 1 | PINK1 | 4.92E-07 | 0.47 | 3.74 | 7.89 | 0.8 | 0.67 | 0.93 |
| cg25465019 | 1 | LMO4 | 0.00108 | 0.21 | 0.56 | 2.69 | 0.8 | 0.67 | 0.94 |
| cg27552081 | 17 | WSB1 | 0.00546 | 0.5 | 2 | 4.03 | 0.8 | 0.66 | 0.93 |
| cg01228134 | 2 | ECEL1 | 5.25E-07 | 0.47 | 3.7 | 7.83 | 0.79 | 0.65 | 0.93 |
| cg03733219 | 19 | SPRED3 | 1.19E-06 | 0.41 | 2.63 | 6.36 | 0.79 | 0.66 | 0.93 |
| cg04672538 | 17 | ARSG; SLC16A6 | 0.00162 | 0.43 | 1.69 | 3.95 | 0.79 | 0.65 | 0.93 |
| cg05310071 | 17 | PIGL | 0.03541 | 0.48 | 1.34 | 2.82 | 0.79 | 0.66 | 0.93 |
| cg07318050 | 1 | C1orf57 | 0.00128 | 0.48 | 2.16 | 4.54 | 0.79 | 0.65 | 0.92 |
| cg11245569 | 11 | TRIM66 | 2.50E-28 | 0.44 | 19.2 | 44.11 | 0.79 | 0.65 | 0.92 |
| cg11964823 | 6 | MICB | 9.08E-11 | 0.45 | 4.76 | 10.56 | 0.79 | 0.65 | 0.93 |
| cg11995490 | 7 | C7orf50 | 1.74E-28 | 0.49 | 23.59 | 47.79 | 0.79 | 0.65 | 0.93 |
| cg12425861 | 14 | PACS2 | 1.26E-23 | 0.48 | 11.41 | 23.98 | 0.79 | 0.66 | 0.93 |
| cg15313956 | 14 | CCDC88C | 1.40E-27 | 0.46 | 24.61 | 53.01 | 0.79 | 0.65 | 0.93 |
| cg15790941 | 4 | C4orf34 | 0.01443 | 0.5 | 1.76 | 3.52 | 0.79 | 0.65 | 0.92 |
| cg17173767 | 8 | C8orf84 | 0.00026 | 0.42 | 1.96 | 4.61 | 0.79 | 0.65 | 0.92 |
| cg18003214 | 7 | GBX1 | 0.00011 | 0.28 | 1.02 | 3.66 | 0.79 | 0.66 | 0.93 |
| cg19499452 | 14 | PACS2 | 4.16E-29 | 0.3 | 4.98 | 16.38 | 0.79 | 0.66 | 0.93 |
| cg01346114 | 17 | GPS2 | 0.00670 | 0.4 | 1.27 | 3.15 | 0.78 | 0.64 | 0.92 |
| cg01519350 | 3 | ARMC8 | 1.40E-05 | 0.46 | 2.93 | 6.31 | 0.78 | 0.64 | 0.92 |
| cg04217140 | 17 | ARRB2 | 0.01038 | 0.49 | 1.8 | 3.65 | 0.78 | 0.64 | 0.92 |
| cg04514249 | 4 | FREM3 | 2.66E-05 | 0.4 | 2.1 | 5.2 | 0.78 | 0.64 | 0.92 |
| cg04621255 | 9 | ENDOG | 9.90E-05 | 0.5 | 3.03 | 6.07 | 0.78 | 0.64 | 0.92 |
| cg05522774 | 21 | OLIG1 | 0.00677 | 0.47 | 1.75 | 3.7 | 0.78 | 0.64 | 0.92 |
| cg05733554 | 14 | C14orf37 | 0.00282 | 0.4 | 1.39 | 3.47 | 0.78 | 0.63 | 0.92 |
| cg06346696 | 3 | TUSC2 | 0.00163 | 0.45 | 1.85 | 4.13 | 0.78 | 0.64 | 0.92 |
| cg07936541 | 2 | ANKRD36B | 0.00022 | 0.49 | 2.76 | 5.59 | 0.78 | 0.64 | 0.92 |
| cg08862778 | 1 | MTOR | 6.24E-05 | 0.5 | 3.08 | 6.23 | 0.78 | 0.64 | 0.92 |
| cg08957484 | 5 | CCNI2 | 0.00579 | 0.5 | 2.01 | 4.03 | 0.78 | 0.65 | 0.92 |
| cg09282338 | 20 | NXT1 | 0.00018 | 0.42 | 1.96 | 4.69 | 0.78 | 0.65 | 0.92 |
| cg11200917 | 5 | GLRA1 | 0.00065 | 0.46 | 2.1 | 4.6 | 0.78 | 0.64 | 0.92 |
| cg11630226 | 8 | LY6K | 2.00E-19 | 0.49 | 10.26 | 20.96 | 0.78 | 0.65 | 0.92 |
| cg12150111 | 6 | PPP1R3G | 0.00046 | 0.48 | 2.44 | 5.07 | 0.78 | 0.65 | 0.92 |
| cg12441052 | 11 | ZDHHC24; ACTN3 | 6.52E-08 | 0.43 | 3.36 | 7.74 | 0.78 | 0.65 | 0.92 |
| cg12637942 | 11 | NEAT1 | 0.00286 | 0.49 | 2.07 | 4.26 | 0.78 | 0.65 | 0.92 |
| cg13428516 | 19 | MAMSTR; RASIP1 | 3.05E-11 | 0.48 | 5.75 | 12.03 | 0.78 | 0.64 | 0.92 |
| cg14781281 | 6 | HLA-J; NCRNA00171 | 0.00200 | 0.47 | 2 | 4.26 | 0.78 | 0.64 | 0.92 |
| cg14807365 | 17 | SLC5A10; FAM83G | 0.00155 | 0.5 | 2.33 | 4.7 | 0.78 | 0.64 | 0.92 |
| cg15047889 | 8 | FAM91A1 | 0.00220 | 0.48 | 2.06 | 4.3 | 0.78 | 0.64 | 0.92 |
| cg16104283 | 1 | SDC3 | 0.00212 | 0.45 | 1.82 | 4.04 | 0.78 | 0.65 | 0.92 |
| cg16943151 | 10 | RHOBTB1 | 2.87E-28 | 0.45 | 20.46 | 45.64 | 0.78 | 0.64 | 0.92 |
| cg19021197 | 17 | TBX2 | 0.00078 | 0.5 | 2.5 | 5.04 | 0.78 | 0.64 | 0.92 |
| cg20039944 | 12 | TRIAP1; GATC | 0.03456 | 0.43 | 1.12 | 2.59 | 0.78 | 0.64 | 0.92 |
| cg21088281 | 4 | GPM6A | 0.00051 | 0.47 | 2.28 | 4.86 | 0.78 | 0.65 | 0.92 |
| cg25432323 | 16 | AARS | 0.01883 | 0.48 | 1.52 | 3.19 | 0.78 | 0.64 | 0.92 |
| cg25969878 | 10 | STK32C | 6.63E-18 | 0.48 | 8.71 | 18.33 | 0.78 | 0.64 | 0.92 |
| cg27588119 | 17 | RNFT1 | 0.03579 | 0.48 | 1.36 | 2.84 | 0.78 | 0.64 | 0.92 |
| cg00259755 | 10 | PWWP2B | 2.65E-09 | 0.5 | 5.35 | 10.79 | 0.77 | 0.63 | 0.92 |
| cg00347643 | 7 | YWHAG | 0.00679 | 0.49 | 1.86 | 3.82 | 0.77 | 0.63 | 0.91 |
| cg01341170 | 16 | SHISA9 | 0.04090 | 0.47 | 1.25 | 2.68 | 0.77 | 0.63 | 0.92 |
| cg02010894 | 19 | CHERP | 0.01191 | 0.44 | 1.38 | 3.14 | 0.77 | 0.63 | 0.91 |
| cg02579136 | 11 | WNT11 | 0.00204 | 0.43 | 1.63 | 3.82 | 0.77 | 0.63 | 0.91 |
| cg02816003 | 6 | RFX6 | 0.03608 | 0.49 | 1.44 | 2.92 | 0.77 | 0.62 | 0.91 |
| cg04330371 | 15 | NR2F2; miR1469 | 1.28E-08 | 0.48 | 4.54 | 9.51 | 0.77 | 0.63 | 0.91 |
| cg04636402 | 5 | NRG2 | 4.35E-11 | 0.46 | 5.25 | 11.32 | 0.77 | 0.63 | 0.91 |
| cg05060949 | 7 | MNX1 | 1.90E-11 | 0.39 | 3.56 | 9.2 | 0.77 | 0.63 | 0.91 |
| cg06248741 | 2 | TXNDC9; EIF5B | 0.00134 | 0.47 | 2.07 | 4.42 | 0.77 | 0.62 | 0.91 |
| cg07082452 | 8 | EGR3 | 1.10E-14 | 0.47 | 7.2 | 15.18 | 0.77 | 0.62 | 0.91 |
| cg08522087 | 5 | ANKH | 2.99E-05 | 0.44 | 2.52 | 5.68 | 0.77 | 0.63 | 0.91 |
| cg09379601 | 19 | DNASE2 | 1.23E-06 | 0.45 | 3.12 | 6.97 | 0.77 | 0.63 | 0.91 |
| cg09858777 | 16 | NUDT16L1 | 0.00265 | 0.44 | 1.65 | 3.79 | 0.77 | 0.63 | 0.92 |
| cg13951491 | 1 | HPDL | 5.04E-13 | 0.44 | 5.18 | 11.89 | 0.77 | 0.63 | 0.92 |
| cg14162940 | 20 | C20orf160 | 9.27E-09 | 0.47 | 4.41 | 9.39 | 0.77 | 0.63 | 0.91 |
| cg14219599 | 6 | GNL1; PRR3 | 0.00782 | 0.45 | 1.51 | 3.39 | 0.77 | 0.63 | 0.92 |
| cg16678169 | 2 | ALS2CR4 | 1.09E-30 | 0.36 | 8.41 | 23.47 | 0.77 | 0.63 | 0.91 |
| cg19496491 | 11 | TEAD1 | 1.62E-06 | 0.48 | 3.54 | 7.44 | 0.77 | 0.63 | 0.91 |
| cg20358834 | 11 | LRFN4; PC | 0.01857 | 0.42 | 1.16 | 2.79 | 0.77 | 0.63 | 0.91 |
| cg22753607 | 9 | ZCCHC7 | 0.02120 | 0.46 | 1.37 | 2.99 | 0.77 | 0.62 | 0.91 |
| cg23279021 | 5 | TMEM232 | 1.58E-15 | 0.49 | 8.43 | 17.12 | 0.77 | 0.63 | 0.91 |
| cg25030018 | 4 | STATH | 1.68E-28 | 0.4 | 8.52 | 21.48 | 0.77 | 0.62 | 0.91 |
| cg25204764 | 1 | SRRM1 | 9.32E-29 | 0.5 | 22.55 | 45.55 | 0.77 | 0.63 | 0.92 |
| cg02841941 | 3 | P2RY1 | 0.00058 | 0.47 | 2.29 | 4.86 | 0.76 | 0.62 | 0.91 |
| cg03531853 | 9 | KIF27 | 5.91E-17 | 0.39 | 4.97 | 12.86 | 0.76 | 0.61 | 0.9 |
| cg07055616 | 10 | NKX6-2 | 0.01553 | 0.48 | 1.57 | 3.3 | 0.76 | 0.62 | 0.91 |
| cg08304084 | 16 | SALL1 | 5.44E-28 | 0.48 | 24.75 | 51.21 | 0.76 | 0.61 | 0.9 |
| cg08526825 | 16 | SRRM2; LOC100128788 | 0.00970 | 0.44 | 1.43 | 3.25 | 0.76 | 0.61 | 0.9 |
| cg09315468 | 8 | DDHD2 | 0.00013 | 0.38 | 1.65 | 4.37 | 0.76 | 0.62 | 0.9 |
| cg10543501 | 5 | HAND1 | 3.38E-07 | 0.45 | 3.32 | 7.43 | 0.76 | 0.62 | 0.91 |
| cg13269439 | 11 | SF3B2 | 0.01431 | 0.5 | 1.74 | 3.5 | 0.76 | 0.62 | 0.91 |
| cg13390975 | 5 | BRIX1; RAD1 | 1.28E-08 | 0.49 | 4.86 | 9.91 | 0.76 | 0.61 | 0.9 |
| cg14172283 | 9 | TOMM5 | 0.04748 | 0.44 | 1.06 | 2.42 | 0.76 | 0.61 | 0.9 |
| cg15243856 | 20 | MATN4; RBPJL | 1.57E-10 | 0.5 | 6 | 12.09 | 0.76 | 0.62 | 0.9 |
| cg17351385 | 19 | ALKBH6 | 0.02716 | 0.49 | 1.5 | 3.07 | 0.76 | 0.62 | 0.9 |
| cg19754622 | 10 | STK32C | 1.39E-28 | 0.44 | 18.92 | 42.69 | 0.76 | 0.62 | 0.9 |
| cg19859486 | 3 | SACM1L | 0.00160 | 0.5 | 2.31 | 4.67 | 0.76 | 0.62 | 0.91 |
| cg21126573 | 17 | KDM6B | 0.00956 | 0.41 | 1.24 | 3.03 | 0.76 | 0.61 | 0.9 |
| cg22577136 | 1 | IKBKE | 0.04066 | 0.47 | 1.3 | 2.73 | 0.76 | 0.62 | 0.9 |
| cg22831315 | 13 | SPG20 | 0.02678 | 0.49 | 1.51 | 3.09 | 0.76 | 0.61 | 0.9 |
| cg23514016 | 5 | BHMT | 0.00053 | 0.49 | 2.57 | 5.2 | 0.76 | 0.61 | 0.9 |
| cg25010788 | 1 | NKAIN1 | 1.43E-12 | 0.5 | 7.19 | 14.39 | 0.76 | 0.62 | 0.91 |
| cg26862691 | 16 | CDK10 | 0.00981 | 0.47 | 1.6 | 3.44 | 0.76 | 0.61 | 0.9 |
| cg01261044 | 14 | SRP54 | 0.02372 | 0.48 | 1.51 | 3.12 | 0.75 | 0.6 | 0.9 |
| cg04840494 | 5 | SERINC5 | 0.03542 | 0.46 | 1.23 | 2.7 | 0.75 | 0.61 | 0.9 |
| cg07650554 | 16 | SEPHS2 | 0.00453 | 0.46 | 1.74 | 3.78 | 0.75 | 0.61 | 0.9 |
| cg07973095 | 16 | DECR2 | 9.97E-12 | 0.44 | 4.82 | 10.98 | 0.75 | 0.61 | 0.9 |
| cg12838902 | 7 | SLC29A4 | 1.03E-08 | 0.47 | 4.47 | 9.45 | 0.75 | 0.61 | 0.9 |
| cg14102128 | 2 | SEPT10; ANKRD57 | 0.03198 | 0.49 | 1.45 | 2.97 | 0.75 | 0.61 | 0.9 |
| cg19436567 | 6 | ARID1B | 0.00786 | 0.48 | 1.75 | 3.67 | 0.75 | 0.6 | 0.9 |
| cg20556702 | 21 | C21orf91 | 1.30E-12 | 0.45 | 5.31 | 11.93 | 0.75 | 0.61 | 0.9 |
| cg21181453 | 9 | DPM2 | 3.53E-27 | 0.5 | 14.8 | 29.74 | 0.75 | 0.61 | 0.9 |
| cg22167789 | 19 | ONECUT3 | 1.59E-05 | 0.46 | 2.92 | 6.28 | 0.75 | 0.6 | 0.9 |
| cg22546168 | 10 | VENTX | 0.00069 | 0.41 | 1.72 | 4.14 | 0.75 | 0.61 | 0.9 |
| cg22793735 | 3 | PLOD2 | 0.04553 | 0.46 | 1.2 | 2.59 | 0.75 | 0.6 | 0.9 |
| cg23664459 | 14 | INSM2 | 4.08E-08 | 0.31 | 1.79 | 5.81 | 0.75 | 0.61 | 0.9 |
| cg27143049 | 11 | PDE3B; PSMA1 | 1.80E-07 | 0.44 | 3.29 | 7.49 | 0.75 | 0.61 | 0.9 |
| ch.2.4639917R | 2 | ARMC9 | 0.01608 | 0.42 | 1.2 | 2.86 | 0.75 | 0.6 | 0.89 |
