# Supplementary material for: Deep Learning/Artificial Intelligence and Blood-Based DNA Epigenomic Prediction of Cerebral Palsy
Source: Int J Mol Sci. 2019 Apr 27;20(9):2075. doi: 10.3390/ijms20092075 (PMC6539236; doi:10.3390/ijms20092075)
Supplement: Supplementary file 1 [file ijms-20-02075-s001.zip › ijms-437963-supplementary/6-CP-Supplemental Table S3.docx]

**Supplementary Table S3.** Differential methylation region (DMR) analysis showing the genomic co-ordinates and overlapping promoters significantly associated with cerebral palsy.

| Sl No. | Chr | Start  co-ordinate | End  co-ordinate | CpGs (N) | FDR | Mean beta fold change | Overlapping Promoters |
| --- | --- | --- | --- | --- | --- | --- | --- |
| 1 | chr6 | 32120324 | 32121611 | 36 | 3.38E-41 | 0.068 | ATF6B-007, ATF6B-006 |
| 2 | chr11 | 2292361 | 2293593 | 32 | 2.52E-39 | 0.039 | NA |
| 3 | chr6 | 31733619 | 31734580 | 18 | 1.49E-52 | 0.064 | CLIC1-004, CLIC1-001, CLIC1-202 |
| 4 | chr6 | 30094960 | 30095802 | 25 | 4.29E-35 | 0.049 | NA |
| 5 | chr6 | 32163966 | 32164927 | 25 | 2.62E-32 | 0.016 | EGFL8-002, EGFL8-001, EGFL8-004, EGFL8-003, EGFL8-005 |
| 6 | chr6 | 30166423 | 30166810 | 17 | 3.86E-29 | 0.021 | TRIM15-002 |
| 7 | chr15 | 75019070 | 75019376 | 10 | 4.28E-33 | 0.068 | SCAMP5-012 |
| 8 | chr6 | 32134620 | 32135396 | 26 | 1.06E-31 | 0.023 | NA |
| 9 | chr6 | 32055046 | 32055534 | 15 | 3.63E-29 | 0.021 | NA |
| 10 | chr6 | 1.44E+08 | 1.44E+08 | 7 | 8.86E-29 | 0.054 | NA |
| 11 | chr11 | 1.22E+08 | 1.22E+08 | 5 | 2.99E-29 | 0.073 | NA |
| 12 | chr7 | 94286473 | 94286669 | 8 | 8.50E-29 | 0.030 | NA |
