# Supplementary material for: Deep Learning/Artificial Intelligence and Blood-Based DNA Epigenomic Prediction of Cerebral Palsy
Source: Int J Mol Sci. 2019 Apr 27;20(9):2075. doi: 10.3390/ijms20092075 (PMC6539236; doi:10.3390/ijms20092075)
Supplement: Supplementary file 1 [file ijms-20-02075-s001.zip › ijms-437963-supplementary/7-CP-Supplementary Table S4.docx]

**Supplementary Table S4.** Prediction of CP based on 42 genes reportedly differently expressed in blood (van Eyk et al. 2018) and also differentially methylated in this study.

|  | **SVM** | **GLM** | **PAM** | **RF** | **LDA** | **DL** |
| --- | --- | --- | --- | --- | --- | --- |
| **AUC**  **95% CI** | 0.9203  (0.6203–1) | 0.9458  (0.6458–1) | 0.8844  (0.6844–1) | 0.8874  (0.6874–1) | 0.7968  (0.5768–1) | 0.9687  (0.6687–1) |
| **Sensitivity** | 0.8700 | 0.8500 | 0.7200 | 0.8000 | 0.7500 | 0.9644 |
| **Specificity** | 0.8500 | 0.9000 | 0.9000 | 0.8000 | 0.8500 | 0.9000 |

Important predictors in descending order of 42 differentially-expressed genes:

SVM: cg00785170, cg16824301, cg22234080, cg10149021, cg25365050

GLM: cg00785170, cg07516697, cg16824301, cg20509092, cg10149021

PAM: cg00785170, cg20509092, cg11961138, cg16824301, cg10149021

RF: cg00785170, cg22234080, cg10149021, cg16824301, cg20509092

LDA: cg00785170, cg22234080, cg16824301, cg20509092, cg26707202

DL: cg07516697, cg10149021, cg16824301, cg22234080, cg11035303
