# Supplementary material for: Deep Learning/Artificial Intelligence and Blood-Based DNA Epigenomic Prediction of Cerebral Palsy
Source: Int J Mol Sci. 2019 Apr 27;20(9):2075. doi: 10.3390/ijms20092075 (PMC6539236; doi:10.3390/ijms20092075)
Supplement: Supplementary file 1 [file ijms-20-02075-s001.zip › ijms-437963-supplementary/8-CP Supplementary Table S5.docx]

**Supplementary Table S5.** IPA canonical pathway statistics.

| **Canonical Pathways** | ***p*-value** |
| --- | --- |
| Crosstalk between Dendritic Cells and Natural Killer Cells | 1.25E-03 |
| Neuroinflammation Signaling Pathway | 5.69E-02 |
| Actin Cytoskeleton Signaling | 2.00E-03 |
| Axonal Guidance Signaling | 2.74E-02 |
| Tight Junction Signaling | 5.06E-02 |
| Insulin Receptor Signaling | 3.93E-02 |
| TGF-B Signaling | 4.41E-02 |
| PI3K/AKT Signaling | 2.84E-02 |
| Neuregulin Signaling | 4.02E-02 |
| Ephrin Receptor Signaling | 4.69E-02 |
