# Supplementary material for: Deep Learning/Artificial Intelligence and Blood-Based DNA Epigenomic Prediction of Cerebral Palsy
Source: Int J Mol Sci. 2019 Apr 27;20(9):2075. doi: 10.3390/ijms20092075 (PMC6539236; doi:10.3390/ijms20092075)
Supplement: Supplementary file 1 [file ijms-20-02075-s001.zip › ijms-437963-supplementary/9-CP Supplementary Methods.docx]

**Supplementary Artificial Intelligence (AI) Methods**

**Artificial Intelligence Analysis Method Data Preprocessing**

The logged value of each CpG was centered by its beta values and auto scaled by its standard deviation (s) to regulate the balance between high and low-intensity features, and to moderate the heteroscedasticity. We utilized quantile normalization to diminish sample-to-sample difference.

**Deep Learning**

Classical machine learning techniques make predictions directly from a set of features that have been pre-specified by the user. However, representation learning techniques transform features into some intermediate representation prior to mapping them to final predictions. DL is a form of representation learning that uses multiple transformation steps to create very complex features [1].

DL is categorized into feed-forward artificial neural networks (ANNs), with multiple layers between the input and output layers. DL finds the correct mathematical manipulation to turn the input into the output, whether the relationship is linear or non-linear. The network propagates through the layers while calculating the probability of each output. First, the DL creates a map of virtual neurons and assigns random numerical values, or "weights", to connections between them. The weights and inputs are multiplied and yield an output value between ‘0’ and ‘1’. If the network does not recognize a particular pattern, an algorithm adjusts the weights. That way the algorithm can make certain parameters more influential, until it determines the correct mathematical manipulation to fully process the data.

In the DL model, more than one hidden layer (y) connect the input (x) and output layer (z) via a weight (W) matrix. We calculated the activation value of the hidden layer (y) by the sigmoid of the multiplication of the input sample ‘x ‘with the weight matrix value ‘W’ and bias ‘b’. The transpose of the weight matrix W and the bias b were then used to construct the output (z) layer. The optimal set of the weight matrix W and bias b are chosen in order to minimize the difference between the input layer (x) and the output layer (z) [2].

To start, the first hidden layer (y) was activated by providing the sample input (x) to the first layer and deciding on the best parameters (W, b). Then, the second layer was predicted by utilizing first hidden layer (y). The same process was repeated for all remaining layers-updating the weights and bias for each layer. Subsequently, we used back-propagation to regulate the parameters for all hidden layers. Finally, the Softmax classifier was used for the final hidden layer to assign new labels to the samples. We used the h2o R computer package [3] to tune the parameters of the DL model.

**Other Machine Learning Algorithms**

In addition to DL we also evaluated a representative set of five ML algorithms which have been applied to metabolomics and genomics data for classification and regression analyses [2]. Below is a synopsis of each of these ML approaches:

**Random forest (RF)** is a widely used machine learning algorithm based on decision tree theory. It works well for high-dimensional data and can accommodate unbalanced and missing values in the data set.

**Support vector machine (SVM)** is another machine learning algorithm that has been used to separate Over-represented canonical pathways, biological processes and molecular processes was identified. Over-represented canonical pathways, biological processes and molecular processes was identified.

**Linear discriminant analysis (LDA)** is closely related to analysis of variance (ANOVA) and regression analysis, and is used to express one dependent variable as a linear combination of other features or measurements.

**Prediction analysis for microarrays (PAM)** has been used to perform sample classification from gene expression data.

**Generalized linear model (GLM)—**logistic regression—measures the relationship between the categorical dependent variable and one or more independent variables by estimating probabilities using a logistic function, which is the cumulative logistic distribution.

To get the optimal predictive performance, we used the caret R computer package [4] to tune the parameters in the models.

**Modeling and Evaluation**

Outcomes prediction was based on methylation levels of CpG loci (epigenomic analysis). Predictive accuracy was assessed based on area under the receiver-operating characteristic ROC curve [AUC (95% CI] along with sensitivity and specificity values.

We randomly split the data into an 80% training set and the 20% as the test set. We chose this ratio to help guarantee a sufficient number of training samples to build a robust model while having adequate number of testing samples to evaluate the model. We performed 10-fold cross-validation (CV) on the 80% training data during the model construction process and tested the model on the hold out 20% of data. We used the R package, pROC, to compute area under the curve (AUC) of a receiver-operating characteristic (ROC) curve to assess the overall performance of the models. To avoid bias, we repeated the above described splitting process 10 times and calculated the average AUC on the 10 hold out test sets. The AUC and the 95% CI along with sensitivity and specificity values were calculated for each of the different AI techniques including DL and also an AI based on regression techniques.

**CP Prediction Based on AI Analysis**

The area under the ROC curve (95% CI), sensitivity and specificity were calculated based on the top 240 best performing individual CpG loci (based on individual AUC, fold-change in methylation, and absolute percentage methylation difference and FDR *p*-value for CP versus controls). This was repeated using only the 76 individual loci that exceeded the stringency threshold. i.e.. *p*-value < 5 × 10^–8^. Finally, we identified differentially expressed genes in leucocytes of CP cases compared to controls [5] which also demonstrated significantly differentially methylated CpG locus/loci in our study and evaluated their predictive accuracy for CP detection.

The following parameters were used to tune the DL model:

• Epochs (number of passes of the full training set),

• l1 (penalty to converge the weights of the model to 0),

• l2 (penalty to prevent the enlargement of the weights),

• Input dropout ratio (ratio of ignored neurons in the input layer during training),

• Number of hidden layers;

The parameters that were used to tune the SVM model was the cost of classification; to tune the RF model was the number of trees to fit; to tune the PAM model was the threshold amount for shrinking toward the centroid.

**Overfitting and Computation Time**

Two common challenges with the use of DL are overfitting and the computation time. DL is prone to overfitting because of the added layers of abstraction, which allows it to model rare dependencies in the training data. To avoid overfitting in the DL model, we used three regularization parameters: L1, which increases model stability and causes many weights to become 0 and L2, which prevents weight enlargement. L1 lets only strong weights survive (constant pulling force towards zero), while L2 prevents any single weight from getting too. Dropout [6] has recently been introduced as a powerful generalization technique, and is available as a parameter per layer, including the input layer. The key idea is to randomly drop units (along with their connections) from the neural network during training. This prevents units from excessive co-adapting. The third parameter that we used for avoiding overfitting in DL model was the input dropout ratio which controls the amount of input layer neurons that are randomly dropped (set to zero) and controls overfitting with respect to the input data. This is particularly useful for high-dimensional noisy data.

**Feature Importance**

Feature (predictor) importance was estimated using a model-based approach. A feature is considered important if it contributes to the model performance. We used the variable importance functions in h2o (varimp) and in caret R packages (varimp) to rank the models features in each of the predictive algorithms.

**Sample Source Code of AI-ML/DL Models**

#The below pseudo code compares the performance AUC of the deep learning (implemened in H2O R #package) and five other different algorithms implemented in CARET R package.

**#Load libraries**

library(mlbench)
library(caret) # six machine learning algorithms

library(h2o) #Deep learning algorithm

library(pROC) # AUC plot

library(preprocessCore) #Quantile normalization

library(RFmarkerDetector) #Scalling

library(ggplot2)# Plot of the AUC

**#Load the dataset**

prostate_df <- read.csv(file="CP_Dif_42.csv",check.names = T, stringsAsFactors = TRUE)

**#Remove Sample Lables from the dataset**

prostate_df <- prostate_df[,-1]# reve sampel labels

prostate_df$subtype <- as.factor(ifelse(prostate_df$subtype==1,1,0))

**#Preprocessing and Quantile Normalization**

metadatanorm=normalize.quantiles(t(as.matrix(prostate_df[,-ncol(prostate_df)])))[, ncol(prostate_df)]

preprocessParams <- preProcess(prostate_df[,1:ncol(prostate_df) -1], method=c("center", "scale"))

**#Randomly Split the data into training and testing**

**#Training and tunning parameters**

#DL Parameters: Epochs, l1 , l2, input dropout ratio, number of hidden layers

#SVM Parameter: Cost of classification

#RF Parameter: the number of trees

#PAM Parameter: the threshold amount for shrinking toward the centroid

#This code is an example for RF Model, the others were implemented very similarly

for (k in 1:10) {

###############Shuffle stat first

rand <- sample(nrow(prostate_df))

prostate_df=prostate_df[rand, ]

###############Randomly Split the data in to training and testing

trainIndex <- createDataPartition(prostate_df$subtype, p = .8,list = FALSE,times = 1)

irisTrain <- prostate_df[ trainIndex,]

irisTest <- prostate_df[-trainIndex,]

irisTrain$subtype=as.factor(paste0("X",irisTrain$subtype))

irisTest$subtype=as.factor(paste0("X",irisTest$subtype))

################################Training and tunning parameters

# prepare training scheme

control <- trainControl(method="cv", number=10,classProbs = TRUE,summaryFunction =

twoClassSummary)

#RF ALGORITHM

set.seed(8)

fit.rf <- train(subtype~., data=irisTrain, method="rf", trControl=control,metric="ROC")

performance_training[1,1]=max(fit.rf$results$ROC) #AUC

performance_training[2,1]=fit.rf$results$Sens[which.max(fit.rf$results$ROC)]# sen

performance_training[3,1]=fit.rf$results$Spec[which.max(fit.rf$results$ROC)]# spec

importance <- varImp(fit.rf, scale=FALSE)

# summarize importance

print(importance)

# plot importance

plot(importance)

**Model Testing**

#Model Testing

rfClasses <- predict( fit.rf, newdata = irisTest,type="prob")

rfClasses1 <- predict( fit.rf, newdata = irisTest)

rfConfusion=confusionMatrix(data = rfClasses1, irisTest$subtype)

rf.ROC <- roc(predictor=rfClasses$X1,response=irisTest$subtype,levels=rev(levels(irisTest$subtype)))

performance_testing[1,1]=as.numeric(rf.ROC$auc)#AUC

performance_testing[2,1]=rfConfusion$byClass[1]#SENS

performance_testing[3,1]=rfConfusion$byClass[2]#SPEC

performance_testing[4,1]=rfConfusion$overall[1]#accuracy

performance_testing[5,1]=rfConfusion$byClass[5]#precision

performance_testing[6,1]=rfConfusion$byClass[6]#recall = sens

performance_testing[7,1]=rfConfusion$byClass[7]#F1

performance_testing[8,1]=rfConfusion$byClass[11]#BALANCED ACCURACY

performance_testing_list[[k]]=performance_testing

performance_training_list[[k]]=performance_training

performance_training=matrix( rep( 0, len=3), nrow = 3) #AUC SENS SPECF

performance_testing=matrix( rep( 0, len=8), nrow = 8) # ROC SENS SPEC

}

**# DL ALGORITHM**

prostate.hex<-as.h2o(irisTrain, destination_frame="train.hex")

#Model Testing

hyper_params <- list(

activation=c("Rectifier","Tanh"),

hidden=list(c(100),c(200),c(10,10),c(20,20),c(50,50),c(30,30,30),c(25,25,25,25)),

input_dropout_ratio=c(0,0.05,0.1),

l1=seq(0,1e-4,1e-6),

l2=seq(0,1e-4,1e-6),

train_samples_per_iteration =c(0,-2),

epochs = c(500),

variable_importances=T,

momentum_start=c(0,0.5),

rho=c(0.5,0.99),

quantile_alpha=c(0,1),

huber_alpha=seq(0,1) )

search_criteria = list(strategy = "RandomDiscrete", max_models = 100, stopping_rounds=5,

stopping_tolerance=1e-2)

dl_random_grid <- h2o.grid(

algorithm="deeplearning",

grid_id = "dl_grid_randome1",

training_frame=prostate.hex,

x=predictors,

y="subtype",

seed=1,

variable_importances=TRUE,

export_weights_and_biases=T,

standardize=T,

stopping_metric="misclassification",

stopping_tolerance=1e-2, ## stop when logloss does not improve by >=1% for 2 scoring events

stopping_rounds=2,

score_duty_cycle=0.025, ## don't score more than 2.5% of the wall time

hyper_params = hyper_params,

search_criteria = search_criteria,

nfolds=2

)

grid <- h2o.getGrid("dl_grid_randome1",sort_by="mse",decreasing=FALSE)

grid@summary_table[1,]

best_model <- h2o.getModel(grid@model_ids[[1]]) ## model with lowest logloss

h2o.varimp_plot(best_model, num_of_features = NULL)

performance_training[1,1]=as.numeric(best_model@model$cross_validation_metrics_summary$mean)[2] #AUC

performance_training[2,1]=as.numeric(best_model@model$cross_validation_metrics_summary$mean)[17]# sen

performance_training[3,1]=as.numeric(best_model@model$cross_validation_metrics_summary$mean)[19]# spec

perf=h2o.performance(best_model,as.h2o(irisTest, destination_frame="test.hex"))

performance_testing[1,1]=as.numeric(h2o.auc(perf,h2o.find_threshold_by_max_metric(perf,"f1"))[[1]])#AUC

performance_testing[2,1]=as.numeric(h2o.sensitivity(perf,h2o.find_threshold_by_max_metric(perf,"f1"))[[1]])#SENS

performance_testing[3,1]=as.numeric(h2o.specificity(perf,h2o.find_threshold_by_max_metric(perf,"f1"))[[1]])#SPEC

performance_testing[4,1]=as.numeric(h2o.accuracy(perf,h2o.find_threshold_by_max_metric(perf,"f1"))[[1]])#accuracy

performance_testing[5,1]=as.numeric(h2o.precision(perf,h2o.find_threshold_by_max_metric(perf,"f1")[[1]]))#precision

performance_testing[6,1]=as.numeric(h2o.sensitivity(perf,h2o.find_threshold_by_max_metric(perf,"f1"))[[1]])#recall = sens

performance_testing[7,1]=as.numeric(h2o.F1(perf,h2o.find_threshold_by_max_metric(perf,"f1"))[[1]])#F1

performance_testing[8,1]=(performance_testing[2,1]+performance_testing[3,1])/2 #BALANCED ACCURACY

performance_testing_list[[k]]=performance_testing

performance_training_list[[k]]=performance_training

performance_training=matrix( rep( 0, len=3), nrow = 3) #AUC SENS SPECF

performance_testing=matrix( rep( 0, len=8), nrow = 8) # ROC SENS SPEC

}

**AUC Plot Testing**

######################AUC plot_testing

list_test=performance_testing_list

list_train=performance_training_list

ee=lapply(list_test, function(x) x[1,])

output <- do.call(rbind,lapply(ee,matrix,ncol=1,byrow=TRUE))

AUC_mean=apply(output,2,mean)

AUC_mean=data.frame(value=AUC_mean,Algorithm=c('RF'))

#colnames(AUC_mean)=c('SVM')

pdf("AUC_mean_testing.pdf")

p<-ggplot(data=AUC_mean, aes(x=Algorithm, y=value)) + geom_bar(stat="identity")

plot(p)

dev.off()

**References**

1. Witten, I. H.; Frank, E.; Hall, M. A.; Pal, C. J., *Data Mining, Fourth Edition: Practical Machine Learning Tools and Techniques*. Morgan Kaufmann Publishers Inc.: 2016; p 654.

2. Alakwaa, F. M.; Chaudhary, K.; Garmire, L. X., Deep Learning Accurately Predicts Estrogen Receptor Status in Breast Cancer Metabolomics Data. *J Proteome Res* **2018,** 17, (1), 337-347.

3. Candel, A.; Parmar, V.; LeDell, E.; Arora, A., *Deep Learning with H2O*. 2018.

4. Kuhn, M., Building Predictive Models in R Using the caret Package. . *Journal of Statistical Software* **2008,** 28, (5), 1-26.

5. van Eyk, C. L.; Corbett, M. A.; Gardner, A.; van Bon, B. W.; Broadbent, J. L.; Harper, K.; MacLennan, A. H.; Gecz, J., Analysis of 182 cerebral palsy transcriptomes points to dysregulation of trophic signalling pathways and overlap with autism. *Transl Psychiatry* **2018,** 8, (1), 88.

6. Srivastava, N.; Hinton, G.; Krizhevsky, A.; Sutskever, I.; Salakhutdinov, R., Dropout: a simple way to prevent neural networks from overfitting. *J. Mach. Learn. Res.* **2014,** 15, (1), 1929-1958.
